# Neurological effects of glucocerebrosidase gene mutations

**DOI:** 10.1111/ene.13837

**Published:** 2018-12-13

**Authors:** S. Mullin, D. Hughes, A. Mehta, A. H. V. Schapira

**Affiliations:** ^1^ Department of Clinical Neuroscience UCL Institute of Neurology London UK; ^2^ Institute of Translational and Stratified Medicine University of Plymouth School of Medicine Plymouth UK; ^3^ LSD Unit/Department of Haematology Institute of Immunity and Transplantation UCL London UK

**Keywords:** Gaucher, GBA, glucocerebrosidase, neuronopathic, Parkinson

## Abstract

The association between Gaucher disease (GD) and Parkinson disease (PD) has been described for almost two decades. In the biallelic state (homozygous or compound heterozygous) mutations in the glucocerebrosidase gene (GBA) may cause GD, in which glucosylceramide, the sphingolipid substrate of the glucocerebrosidase enzyme (GCase), accumulates in visceral organs leading to a number of clinical phenotypes. In the biallelic or heterozygous state, GBA mutations increase the risk for PD. Mutations of the GBA allele are the most significant genetic risk factor for idiopathic PD, found in 5%–20% of idiopathic PD cases depending on ethnicity. The neurological consequences of GBA mutations are reviewed and the proposition that GBA mutations result in a disparate but connected range of clinically and pathologically related neurological features is discussed. The literature relating to the clinical, biochemical and genetic basis of GBA PD, type 1 GD and neuronopathic GD is considered highlighting commonalities and distinctions between them. The evidence for a unifying disease mechanism is considered.

## Introduction

Gaucher disease (GD) is an autosomal recessive disorder caused by mutations of the glucocerebrosidase gene (GBA). The GBA encodes the lysosomal hydrolase glucocerebrosidase (GCase), which under acidic conditions will hydrolyse the sphingolipid waste product glucosylceramide into ceramide. Mutations cause a reduction or complete loss of GCase activity which (in the case of some biallelic subjects) leads to the accumulation of glucosylceramide (and its deacylated derivative glucosylsphingosine) in visceral organs, causing a variety of clinical phenotypes. These include thrombocytopaenia, anaemia, hepatosplenomegaly and osteopaenia/osteonecrosis 1, 2. A subset of these patients will also develop central nervous system (CNS) features 1.

Gaucher disease is broadly categorized into three subtypes. Type 1 GD (GD1) is diagnosed in the absence of central neurological features, although peripheral nervous system involvement in the form of a symmetrical polyneuropathy does occur 3. Type 2 GD (GD2) is characterized by rapid neurological deterioration and is ultimately fatal whilst in type 3 GD (GD3) there are slower progressive neurological features. In practice, the distinction between GD2 and GD3 are often blurred and it has been suggested that the exact neurological phenotype is more likely to be a spectrum disorder 4. For the purpose of this review, types 2 and 3 will be referred to collectively as neuronopathic Gaucher disease (nGD).

## The neurological phenotype of nGD

There is a substantial variation in the neurological features associated with nGD. A supranuclear gaze palsy is the most common clinical presentation; however, this may be because it is an easily identified clinical feature which allows the diagnosis to be readily made 1, 4, 5, 6. This advances the question whether in fact the definition of nGD is an arbitrary waypoint in a spectrum of neurological features present across nearly all Gaucher patients 7.

Otherwise cognition is the second most common feature of nGD 5 in combination with a variety of other features such as tremor, myoclonus, seizures, gait disorders, bulbar dysfunction, stridor, muscle weakness 1. There appears to be some regional variation between this phenotype 3, 8, 9, 10, which may or may not be explained by variation in GBA mutations present 11. Equally, attempts to show mutation‐specific phenotypes have been unsuccessful 4, 7.

## Central nervous system features in GD1

A minority of GD1 patients are known to develop peripheral neuropathy 3; however, the prevailing view has been that GD1 patients do not develop central nervous system (CNS) involvement. In recent years, there have been grounds to consider the need for revision of this view. The discovery that GD1 patients have a lifetime risk of developing Parkinson disease (PD) of approximately 10%–30% compared to approximately 1%–2% in the general population indicates that CNS dysfunction as a consequence of a GBA mutation can be lifelong. A study of the cognitive profile of Gaucher patients found that, compared to age‐matched controls, there was delayed recall of verbal and non‐verbal information along with reduced attention 8.

Our own prospective data have shown general cognitive impairment along with impaired olfaction amongst not only GD1 patients but also ‘asymptomatic’ heterozygous GBA carriers 12, 13. Moreover, deterioration of cognition, olfaction and depression was more marked amongst both these groups compared to controls. It is notable that the distribution of these features correlates with the sequence of prodromal symptoms that characterize the preclinical phase of PD.

## Glucocerebrosidase gene mutations and PD

Although a family history of idiopathic PD results in approximately a fourfold increase in lifetime risk 14, it is not ordinarily passed on in a Mendelian manner. The recognition that GBA mutations are an important risk factor for PD has proven to be an important stimulus for additional insights into the pathogenesis of PD. Unlike other genetic forms of PD, GBA mutations display incomplete lifetime penetrance of around 10%–30% 3, 4, 7. The GBA mutation minor allele frequency is 1% in the general population 12, 13, 5% in the Ashkenazi population 3, 15, 16, between 5% and 10% in idiopathic PD 8 and between 10%–30% in Ashkenazi Jews with PD 3, 15, 16. The precise frequencies in the respective populations depend upon whether the whole exome has been sequenced, whether targeted microarray genotyping of specific (common) mutations is carried out and upon the inclusion or not of mutations such as E326K which is not associated with Gaucher disease 12, 13 but is associated with an increased risk of PD 17.

## The neurological phenotype of GBA PD

Although there is some debate about the GBA PD phenotype, it appears to be associated with a more pronounced cognitive deficit. This is supported by data which implicate GBA in dementia with Lewy bodies, a form of dementia with associated parkinsonism and other differentiating features such as hallucinations 18. Equally there are some data to suggest that there is an enhanced neuropsychiatric phenotype. Neuroanatomically, this has been considered to suggest a more ubiquitous spread of Lewy pathology across cortical brain regions, in contrast to PD which predominantly affects the basal ganglia, although increasingly it is accepted that Lewy deposits affect a variety of brain regions, especially in late idiopathic PD 19, 20. That said, asymmetrical reductions in dopamine uptake have been demonstrated amongst GBA PD subjects within the basal ganglia 21.

The clinical course of GBA PD has conclusively been shown to be more aggressive, with a younger age of onset compared to idiopathic PD and a lower median survival time from diagnosis 22. Some data indicate that classical features of PD including asymmetrical onset, bradykinesia, postural instability and rigidity are less pronounced in GBA PD subjects 23. To date, no genetic association has been found with other synucleinopathies such as multisystem atrophy 3.

## Mechanistic explanations for nGD and GBA PD

There is no consensus on the underlying mode of action of neurodegeneration in nGD and GBA PD. It is beyond the scope of this paper to review these data in detail; however, broadly they can be categorized into those which postulate a loss of function and those which postulate a gain of function. The argument of a loss of function centres primarily on the finding of reduced GCase catalytic activity in both GD (blood) and GBA PD [blood and cerebrospinal fluid (CSF)] 11, 24, 25. However, there are major inconsistencies in this argument. In particular, there is a relatively poor correlation between GCase activity levels and disease risk in PD and GD. For instance, one study showed that GCase activity from peripheral blood spots categorized by mutation did not correlate with the severity of those individual mutations in terms of PD. L444P PD cases for instance appear to have higher GCase activity than those with N370S, even though L444P mutations convey between two and three times the risk of PD compared to N370S 11, 26, 27. Equally, heterozygous carriers of the 84GG mutation, which is a frameshift mutation and would be expected to be null in terms of GCase activity, had comparable enzyme activity with other missense GBA mutations 28, 29. Although in GD residual GCase activity has an influence, it does not in isolation predict disease prognosis 28. A number of mechanisms have been proposed by which loss of function could lead to GD and PD. Predominantly, these are accumulation of enzyme substrate (GD and PD) and failure of autophagic pathways leading to reduced disposal of alpha synuclein 30.

It may be that this disparity is a reflection of the limitations of the GCase activity assay. In leucocytes GCase is typically normalized to the protein concentration of the lysate produced and in CSF there is no normalization to protein concentration at all 25, 31. Differences in the expression levels of the mutant and wild‐type GCase protein (from the remaining non‐mutated allele) will substantially influence what is effectively an assay that measures the rate of substrate catalysis, yet to date there is no mechanism to normalize for GCase protein expression.

Conversely, some argue that GBA induces a toxic gain of function. Perhaps the most convincing of these explanations comes from the work of Mia Horowitz who suggests that sequestration of mutant GBA within the endoplasmic reticulum, due to a failure of the normal process of post‐translational folding, leads to a variable degree of unfolded protein response which correlates with the pathogenicity of the mutation 32.

There may of course be the potential for both loss of function and a toxic gain of function to be coexistent or perhaps more plausibly a product of one another. For instance, any structural alteration to the GCase protein may give rise to gain in function but may incidentally reduce enzyme activity. Conversely, a loss of enzyme activity may result downstream in an added gain of function as a result of disruption of the balance of substrate/product.

## Neuropathology of GD and PD

Parkinson disease is pathologically well defined by the degeneration of the substantia nigra pars compacta and the presence of Lewy bodies. These are protein inclusions predominantly comprising alpha synuclein, a ubiquitously expressed cytosolic protein of unknown physiological function that appears to be central to the pathological processes that contribute to PD 33, 34. Its key importance is confirmed by the finding that missense mutations of the alpha synuclein gene (SNCA) cause autosomal dominant familial PD 24, 25 whilst polymorphisms in SNCA cause a marginal protective/causative effect in terms of developing the PD phenotype 11. Interestingly missense mutations in the Rep1 promotor region 11, 26, 27 and duplications/triplication of the whole SNCA gene 28, 29 are also implicated or causative of PD, with the age of onset being proportional to the expression levels of wild‐type alpha synuclein protein. Lewy pathology is present in around 20% of subjects of over 80 years without any signs of parkinsonism, indicating that alpha synuclein accumulates with age, a process that reflects the age‐related incidence of PD 28.

A recent concept in the pathophysiology of PD is that alpha synuclein may be transmitted in a prion‐like fashion 30. Briefly, alpha synuclein is a monomeric protein in the native state; however, under a variety of intracellular conditions (such as proximity to a lipid membrane 35 or oxidative stress 36) it converts to a beta sheet rich fibrillar/oligomeric and then an aggregated form 37. It is this aggregated phosphorylated form that is the major component of Lewy bodies 38. Postmortem examinations of PD patients who received stem cell grafts as part of a clinical trial showed that within a decade transplanted neuronal tissue also contained Lewy pathology 39. It appears that aggregated alpha synuclein can ‘seed’ monomeric forms to adopt these fibrillar/oligomeric/aggregated configurations, which in turn can propagate Lewy pathology to adjacent cells 40, 41, 42, 43.

Some studies have demonstrated Lewy pathology in the enteric nervous system 44, 45, 46 and have suggested that it may propagate along peripheral nerves (most prominently the vagus nerve), through the midbrain and then subsequently into the cortex and neocortex, with PD severity correlating with the extent of the sequential spread of this pathology 47, 48. Concurrently, a series of prodromal features of PD sequentially occur in a manner which is postulated to correlate with the pathological spread 44, 49, 50.

Neuronopathic Gaucher disease has been less well characterized. There are only a few postmortem studies of nGD patients, a substantial portion of which were performed two to three decades ago 51, 52, 53, 54. There have also been a number of mouse models of nGD, although most of these employ artificial disruption of the GBA gene to produce a broadly comparable phenotype and are of questionable clinical relevance 55, 56. A common feature appears to be microgliosis and subsequent astrogliosis and neuronal loss, particularly in hippocampal regions 51, 55, along with substrate accumulation 52, 54, 55, 57, 58. However, there appears to be significant variation in the severity of neuropathology in nGD cases, which is broadly proportional to the degree of CNS involvement 51. A postmortem study of three GD1 patients (all N370S homozygous or N370s compound heterozygous) without clinical neurological involvement revealed microgliosis and astrogliosis in cortical and hippocampal regions 51. This glial activation mimics the distribution of hippocampal pathology in nGD patients, with the distinction that amongst nGD subjects there is accompanying neuronal loss 51.

Recent data suggest that the deacylated derivative of glucosylceramide, glucosylsphingosine, may be the pathogenic molecule in nGD. Most pertinently glucosylsphingosine appears to be raised far more specifically in (plasma) GD subjects than other biomarkers including glucosylceramide 33, 34. Unfortunately, only a few more recent studies have attempted to quantify neuronal levels of the lipid; however, it appears to be present in human nGD 54 and transgenic mouse brains 55.

## Cell biology of GD and GBA PD

Glucocerebrosidase gene mutations may cause aberrant post‐translational folding of the enzyme. This prevents trafficking of the enzyme from the endoplasmic reticulum to the lysosome and causes the GCase to become sequestered within the endoplasmic reticulum 6, 59, 60. The consequences of this are twofold. On the one hand, insufficient GCase reaches the lysosome to allow sufficient catalysis of glucosylceramide to ceramide. The sequestered GCase also gives rise to an unfolded protein response which in turn results in oxidative stress in the cytosol 6, 59, 60, 61.

*In vitro* studies have highlighted a bidirectional inverse correlation between GCase activity and alpha synuclein 62, 63. Concurrently, biophysical studies have shown a direct interaction with alpha synuclein which is lessened for GBA mutation carrying forms of the enzyme 64, 65. These findings are within the broader context of an established literature implicating diminished mechanisms of cellular autophagy in idiopathic PD 66, 67, 68. Interestingly, there appears to be an age‐dependent reduction in GCase activity in both mice and human brain 69, 70. Moreover, GCase activity levels are reduced compared to controls in the brains of non‐GBA idiopathic PD subjects 71; however, studies looking at substrate accumulation are somewhat varied. Some show no increase in glucosylceramide or glucosylsphingosine levels in PD or aged controls 72, 73 but another found an increase in the latter 69. Mouse data have also shown age‐dependent upregulation of glucosylsphingosine and two studies found that ceramide levels (the product of substrate breakdown) are reduced in the brains of GBA PD subjects 74, 75. Thus, although there is evidence to support a role for GCase in the proteolysis of alpha synuclein, with GBA mutations causing reduced GCase activity and in turn reduced turnover of cytosolic alpha synuclein, the precise mechanism underlying this remains unknown.

## Parallels between nGD and GBA PD

### Variable penetrance of nGD and GBA PD

A particularly pertinent aspect of the natural history of nGD and GBA PD is the variable penetrance of the phenotype in both conditions (i.e. many with compatible genotypes will not develop PD, GD1 or nGD). The mutation/phenotype correlation is not absolute, although similar patterns exist across the two conditions. For instance severe GBA mutations, which by definition are associated with nGD 76, increase the risk of PD by around four times compared to mild ones 26. Equally, specific mutations which are known to be highly pathogenic in terms of nGD (such as L444P) 77 are also amongst the most pathogenic in terms of PD risk 78. Conversely those which have a questionable effect on the GD phenotype 24, 25, such as E326K and T369M, deliver only a marginal increase in PD risk 11.

Stratification of disease risk by categorization of mutations has aroused significant interest in the context of PD 11, 26, 27. The severe/mild dichotomy has been applied prospectively to PD cohorts, and it has been shown to have a major impact on age at onset of PD and median time to dementia 28, 29. It may allow more effective targeting of future disease modifying treatments for PD, increase the power of clinical trials of such compounds by allowing selection of subjects who are likely to have a more rapid decline 28. Moreover, the fact that the same mutations determine the severity of neurological features in both GD and PD suggests that a common mode of action is shared by the two conditions.

### Neuroinflammation in GBA PD and nGD

There is evidence for immune dysregulation associated with, and putatively causative of, a number of the major clinical phenotypes of GD 79, 80, 81, 82. Additionally, there is substantial evidence of an increased risk of monoclonal gammopathy, multiple myeloma as well as lymphoid malignancies within GD patients 83, 84. GD patients have been shown to have increased serum concentrations of interleukin 6 and 10 79 whilst GBA knockout mice display upregulation of a variety of immune markers and increased levels of a variety of inflammatory cytokines in the plasma 85, 86. This has been hypothesized to be a result of accumulated glucosylceramide within the mononuclear phagocytic system, although the precise mechanism is still unclear 85.

Immune activation has also been highlighted as a key factor in the pathogenesis of PD 87. Substantial *in vitro* data have directly linked alpha synuclein to microglial activation, with a number of mechanisms postulated 88, 89, 90, 91, 92, 93, 94. This correlates with *in vivo* clinical imaging studies 95, postmortem evidence 96, 97, serum titres of cytokines 98, 99 and autoantibodies to components of the synuclein aggregation pathway 100. Interestingly chronic use of non‐steroidal anti‐inflammatory drugs has been shown to exhibit a mildly protective effect against PD onset 14. One study has also shown enhanced activation of a number of peripheral cytokines within GBA PD patients 101.

## Disease modifying therapy in GD, nGD and GBA PD

### Enzyme replacement therapy

The advent of recombinant enzyme replacement therapy (ERT), given intravenously, usually on an approximately monthly basis, has transformed the prognosis for those with GD 2; the majority of GD1 patients can now live practically normal lives. The exception to this is nGD, which is at present unresponsive to ERT as the 596 kDa protein is unable to cross the blood–brain barrier 2, 102. There are various strategies under consideration which aim to temporarily permeabilize the blood–brain barrier to allow recombinant ERT into the brain 103. These include use of a lentiviral vector to chaperone the protein 104, 105. Intrathecal administration of ERT has been used with some success in paediatric Hurler's syndrome (mucopolysaccharidosis type 1) 106. Moreover, a trial of intrathecal ERT in Hunter's disease (mucopolysaccharidosis type 2) is also under way 107. That said, there are a number of pitfalls which must be overcome. The major challenge is ensuring the ERT is able to breach the cell membrane and reach the intracellular compartment. There is also the very real danger of an immune mediated CNS inflammatory reaction following such administration 106.

### Substrate reduction therapy

Other strategies to overcome the issue of CNS penetrance include substrate reduction therapy. Here inhibition of upstream enzymes in the metabolic pathway such as glucosylceramide synthase actively reduce substrate production. Miglustat is one such compound. Mouse models have shown, using microglial inflammation as a surrogate marker of CNS penetrance, that miglustat crosses the blood–brain barrier 108 and there is some anecdotal evidence of clinical efficacy in GD3. However, a small randomized controlled trial failed to prove this, although this may be on account of the poor choice of primary outcome, namely an improvement in supranuclear gaze palsy 109, 110. Equally its use was associated with an apparently increased incidence of peripheral neuropathy 110, 111.

*In vitro* studies have suggested that, in cell lines expressing autosomal dominant alpha synuclein mutations, these therapies reduce levels of the synuclein protein 17, which has led to a phase II clinical trial of the glucosylceramide synthase inhibitor venglustat as a putative neuroprotective in GBA PD (clinicaltrials.gov, NCT02906020). Whilst intriguing, substrate accumulation has not consistently been demonstrated in PD brains. Nevertheless, it may be that minute undetectable alterations in substrate levels interact with alpha synuclein in other neurotoxic ways, such as for instance by altering the aggregation dynamics of the protein 112.

### Small molecular chaperones

Another therapeutic strategy is the use of small molecular chaperones which are also able to enter the CNS. These compounds appear to modify the tertiary and quaternary structure of GCase, facilitating post‐translational folding and transport of GCase to the lysosome 113. At the lysosome the change in pH causes the chaperone to dissociate from GCase allowing effective catalysis to resume. A recent phase II trial of ambroxol, a repurposed cough linctus which also acts as a small molecular chaperone to GCase, showed good CSF penetrance and a reduction of glucosylceramide levels in the CSF 114. Similarly our own group is currently conducting a phase II trial of ambroxol in PD patients (NCT02941822).

### Gene therapy

Interest in gene therapy targeting the GBA gene up to this point has been predominantly in the context of GD 18. Of particular interest has been the realistic prospect of such therapy crossing the blood–brain barrier and hence being a viable treatment for neurological GBA related disease. Using invariant natural T killer cells, it was shown in 2011 that Gaucher fibroblasts could be transfected using a high‐titre, amphotropic retroviral vector in which human GBA1 was driven by the mutant polyoma virus enhancer/herpesvirus thymidine kinase gene promoter 19, 20. Unfortunately the clinical protocol that resulted achieved a transfection efficiency of only 1%–10% which was insufficient to cause a clinically significant improvement in substrate levels 21. Using a murine GBA knockout model, an intravenously injected lentiviral vector was shown to be able to deliver improved GCase activity, reduced substrate accumulation and improvements in a number of clinical outcomes 22; however, whether such outcomes can be replicated in human subjects is uncertain.

In the context of PD, moreover, it is unclear whether affected brain regions (e.g. the substantia nigra and striatum) could be reached by such vector technologies. However, systemic administration to transgenic synuclein overexpressing mice of AAV9‐PHP.B (an AAV which readily crosses the blood–brain barrier) resulted in complete clearance of alpha synuclein throughout the brain 22. The approach has great promise as a means of readily crossing the blood–brain barrier; however, finding stable and safe means of efficient transfection remains the principal obstacle to its successful clinical application 23.

## Concluding remarks

In this paper, it is suggested that in terms of CNS involvement the traditional dichotomization of GD1 and nGD patients may require revision and that a continuum of neurodegeneration may exist across biallelic and heterozygous carriers of GBA mutations. The evidence for this is mixed. The same (severe) mutations are associated with a worse phenotype in both diseases and the penetrance of this phenotype is similarly variable. Neuropathologically, the presence of substrate deposits in the brains of nGD patients and their low or absent levels in heterozygous GBA PD cases imply no commonality of pathology across the two diseases. However, postmortem studies of nGD and GD1 patients have identified a pattern of microgliosis and then astrogliosis (with varying severity) which is anatomically very similar to that found in dementia with Lewy bodies. In this spectrum GD2 and then GD3 patients represent the more severe neurological phenotypes whilst GD1 patients and heterozygous GBA carriers display variable degrees of mild or subclinical neurological features. PD may simply be another neurological phenotype caused by GBA mutations.

No consensus exists regarding the pathogenic mechanism of nGD or GBA PD; however, a variety of promising disease modifying strategies, targeting a variety of proposed mechanisms, are currently in development or under evaluation. The authors believe that in the near future these approaches may elicit a tangible disease modifying effect in both diseases.

## Disclosure of conflicts of interest

The authors declare no financial or other conflicts of interest.
